# Understanding individual differences in theory of mind via representation of minds, not mental states

**DOI:** 10.3758/s13423-018-1559-x

**Published:** 2019-01-16

**Authors:** Jane R. Conway, Caroline Catmur, Geoffrey Bird

**Affiliations:** 1 MRC Social, Genetic and Developmental Psychiatry Centre, Institute of Psychiatry, Psychology and Neuroscience, King’s College London, London, SE5 8AF UK; 2 Department of Experimental Psychology, University of Oxford, Oxford, OX1 4AL UK; 3 Department of Psychology, Institute of Psychiatry, Psychology & Neuroscience, King’s College London, London, SE1 1UL UK

**Keywords:** Theory of mind, Face-space, Individual differences, Social cognition, Mind-space

## Abstract

The human ability to make inferences about the minds of conspecifics is remarkable. The majority of work in this area focuses on mental state representation (‘theory of mind’), but has had limited success in explaining individual differences in this ability, and is characterized by the lack of a theoretical framework that can account for the effect of variability in the population of minds to which individuals are exposed. We draw analogies between faces and minds as complex social stimuli, and suggest that theoretical and empirical progress on understanding the mechanisms underlying mind representation can be achieved by adopting a ‘Mind-space’ framework; that minds, like faces, are represented within a multidimensional psychological space. This Mind-space framework can accommodate the representation of whole cognitive systems, and may help to explain individual differences in the consistency and accuracy with which the mental states of others are inferred. Mind-space may also have relevance for understanding human development, intergroup relations, and the atypical social cognition seen in several clinical conditions.

## Introduction

Minds, like faces, are a special set of stimuli in the social environment. They are a dynamic source of information about the behavior of conspecifics, with relevance for many aspects of everyday life, from the enjoyment of friendships to how a jury assesses the accused. Understanding how we represent the minds of other humans is therefore a particularly important aim. For the past 27 years, the idea that faces are represented within a multidimensional psychological space has provided a unifying theoretical framework that explains multiple experimental effects and informs new predictions (Valentine, 1991; Valentine, Lewis, & Hills, 2016). The concept of ‘Face-space’ has brought coherence to a large literature, and offers a psychological model of how these multifarious stimuli are processed. In contrast to the literature on face processing, the study of how minds are represented lacks a coherent organizational framework (Happé, Cook, & Bird, 2017).

We suggest that the study of mind representation would benefit from the adoption of a ‘Mind-space’ framework – where minds are represented within a multidimensional space – in the same way as the face processing literature has from the introduction of Face-space (Oosterhof & Todorov, 2008; Todorov, Olivola, Dotsch, & Mende-Siedlecki, 2015; Todorov, Said, Engell, & Oosterhof, 2008; Valentine et al., 2016). We argue that adopting the Mind-space framework would allow explanation of individual differences in the ability to represent minds, and also in the ability to infer mental states. Here, we use the term ‘mind’ to refer to an individual’s complete set of cognitive systems, and the term ‘mental state’ to refer to the representational content generated by that set of systems. The probability of specific mental states is dependent on the properties of the mind to which they are ascribed. Therefore, understanding individual differences in the representation of minds allows individual differences in the accuracy of mental state inference to be explained. For example, the mental state ‘Everyone in the world loves me’ would be more likely to be generated by a mind that has the property of a high degree of narcissism, than one without such a property. Therefore, people who are better able to characterize the specific mind generating a mental state are likely to be more accurate at inferring that mental state. Accordingly, this paper proposes a mechanism by which the ability to represent minds in Mind-space explains skill in accurately inferring mental states.

We outline how the Mind-space framework can enable the following necessary advances: Describe how people represent all properties of minds; explain variance in the quality and structure of such representations; elucidate the processes by which another’s mental states are inferred; and explain individual differences in the accuracy of mental state inference. In order to do so we will make three independent, but related, arguments, namely:

1. Understanding individual differences in representation of mental states is difficult within current frameworks.
2. Although mental states are a product of the individual mind that gave rise to them, representation of minds is largely absent from empirical and theoretical work on mental state inference.
3. Adoption of a Mind-space framework is one way in which representation of minds can be incorporated into the process of mental state inference, and in doing so one can better understand individual differences in mental state inference.

## Understanding individual differences in theory of mind

To date, the study of understanding other minds has focused on how people represent others’ mental states, such as thoughts and beliefs; this ability is most often termed ‘theory of mind’ (Baron-Cohen, Leslie, & Frith, 1985; Premack & Woodruff, 1978). Despite the thousands of studies referencing theory of mind, it is still unclear what individual differences in the ability represent (Bird, 2017; Bartsch & Estes, 1996; Conway & Bird, 2018). This may be due to the lack of theories addressing the underlying psychological processes involved in the representation of mental states (Baker, Jara-Ettinger, Saxe, & Tenenbaum, 2017; Schaafsma, Pfaff, Spunt, & Adolphs, 2015; Spunt & Adolphs, 2015), and how the contents of such representations are derived. Therefore, explanations for individual differences in theory of mind have been limited to invoking domain-general inferential processes such as language (Milligan, Astington, & Dack, 2014) or executive function (Carlson & Moses, 2001; Devine & Hughes, 2014; Hughes, 1998), rather than domain-specific representational structures. Although it is clear that variance in domain-general processes may influence performance on theory of mind tests, variance within these domains would influence performance on most tasks, and variance in such domain-general processes does not inform what it is to be better or worse specifically at representing mental states, and why (Conway & Bird, 2018; Bird, 2017).

Understanding individual differences in theory of mind would be aided by a model of what determines the difficulty of representing different types of mental states *within* an individual. Surprisingly, although there is considerable debate in the literature as to what qualifies as a mental state – for example whether someone’s visual perspective (Samson, Apperly, Braithwaite, Andrews, & Bodley Scott, 2010) or emotional state (Oakley, Brewer, Bird, & Catmur, 2016) qualifies as a mental state, or whether the term should be reserved for representation of propositional attitudes (Butterfill & Apperly, 2013; Leslie, 1987) – there is considerable agreement that certain types of mental state are harder to represent than others. For example, few experts would disagree that it is harder to represent false beliefs (beliefs held by an individual that you know to conflict with reality) than true beliefs (Leslie, 1987; Wimmer & Perner, 1983). Despite this agreement, however, as far as we are aware there is little understanding of what makes some mental states harder to represent than others, beyond the fact that representation of some types of mental state makes greater demands on domain-general processes such as working memory, language, or executive function, than representation of other types of mental state.

In the absence of such understanding, it is important to understand the basis for the consensus of opinion as to the relative difficulty of representing different types of mental state. One important influence is the work of Wellman and colleagues (Shahaeian, Peterson, Slaughter, & Wellman, 2011; Wellman, Fang, Liu, Zhu, & Liu, 2006; Wellman, Fang, & Peterson, 2011; Wellman & Liu, 2004) within the field of developmental psychology. This work has described the developmental trajectory of mental state understanding and noted that understanding of certain types of mental state tends to occur earlier in development than understanding of other types of mental state (e.g., understanding of desires occurs before understanding of beliefs). Such evidence has been used to support the idea that certain types of mental state are more difficult to represent than others. However, the order in which different types of mental state are understood varies across cultures, for instance children in Iran and China tend to understand the relationship between seeing and knowing before appreciating that people can have diverse beliefs, whereas the reverse order is observed in children from Australia and the USA (Shahaeian, Nielsen, Peterson, & Slaughter, 2014a; Shahaeian, Nielsen, Peterson, Aboutalebi, & Slaughter, 2014b; Shahaeian et al., 2011; Slaughter & Perez-Zapata, 2014; Wellman et al., 2006, 2011). This makes it likely that the order in which children understand different types of mental state may instead depend on environmental factors such as when they are taught about each type of mental state (Heyes & Frith, 2014), rather than providing any explanation of, or justification for, differential difficulty of mental state representation (Conway & Bird, 2018; Bird, 2017). Moreover, it is also possible that the age at which children can represent different types of mental state is governed by the degree to which they recruit domain-general processes of executive function or language, and the developmental timetable of these processes (Devine & Hughes, 2014; Milligan et al., 2014; Sabbagh, Xu, Carlson, Moses, & Lee, 2006).

### An absence of minds in tests of theory of mind

Theory of mind is typically defined as the ability to *represent* mental states. In contrast, theory of mind measures tend to test the ability to make accurate mental state *inferences*. This distinction is important; on any particular test one could make an inaccurate mental state inference yet still represent a mental state. In such a situation there is no deficit in the representation of mental states, but rather a deficit in accurately inferring the content of a particular mental state.

Theory of mind tests tend to require the participant to infer the mental state of a protagonist in a certain situation (Baron-Cohen et al., 1985; Dziobek et al., 2006). The ‘correct’ mental state inference is typically determined by the test authors based on rational consensus. Such an operationalization results in a binary response measure: one either can, or cannot, make the correct mental state inference. As a consequence, these measures are not sensitive to subtle variance in the quality of mental state inference processes, and ignore perhaps the most important source of inferential error: representation of the mind giving rise to the mental state.

Specifically, existing tests of mental state inference largely fail to take account of the variability in the populations of minds available for representation, and the degree to which this variability is incorporated into mental state inference. An individual is exposed to many different minds, and ‘mind type’ – the collection of long- and short-term attributes characterizing a particular mind – is likely to influence the kind of mental states a particular mind produces. One can easily imagine that, even in the same objective situation, an optimistic mind may produce very different mental states from a pessimistic mind; an autistic mind different mental states from a neurotypical mind; and an adult mind different mental states from a child’s mind. This variance in mental states as a function of mind type – a crucial component of the accuracy of naturalistic mental state inference – is absent from tests of theory of mind that make use of an anonymous protagonist about whom nothing is known. Even those tests that introduce well-formed characters with distinct personalities, tests that have the potential to examine the degree to which mental state inference varies as a function of the protagonist’s mind type, do not explicitly score this aspect of mental state inference (Dziobek et al., 2006).

Furthermore, although the majority of tests of theory of mind have examined the representation and inference of mental states – the *content* of someone’s mind – there are also multiple *processes* of mind available for representation. The degree to which these are represented, and the accuracy of their representation, is likely to contribute to variance in the accuracy of mental state inference. Several of these mental processes have been addressed by cognitive science, such as memory, attention, and spatial reasoning; but the degree to which they are represented as properties of others’ minds has been less well studied (Camerer, Ho, & Chong, 2004; Coricelli & Nagel, 2009). Moreover, such work has rarely been linked to the representation of other aspects of mind. It is strange that, for example, the evaluation of others’ working memory or metacognitive ability is not linked theoretically to representing their mental states (e.g., thoughts and beliefs), when both constitute properties of another’s mind that are available for representation and which may help predict their subsequent behavior. These processes can be described as features of minds in the same way as personality traits such as optimism or aggressiveness, and may also produce variance in mental states despite an identical situation. A forgetful mind may give rise to different mental states than a mind with good memory; a more intelligent mind may give rise to different mental states than a less intelligent one; and so on. The degree to which individuals incorporate such information in their inference of mental states is also largely untested in current tests of mental state inference.

Without a theoretical framework that addresses variance in other minds and their representation, explanations of individual differences in theory of mind will remain limited to domain-general abilities, rather than the quality of domain-specific representational content and the inferential processes specific to accurate mental state representation. We argue that the development of a theoretical framework that describes representation of whole cognitive systems, i.e. of minds in their entirety, would contribute to the understanding of those psychological processes giving rise to more or less accurate inference of another’s mental states.

## Mind-space: A new framework for understanding the representation of minds

**Fig. 1:**
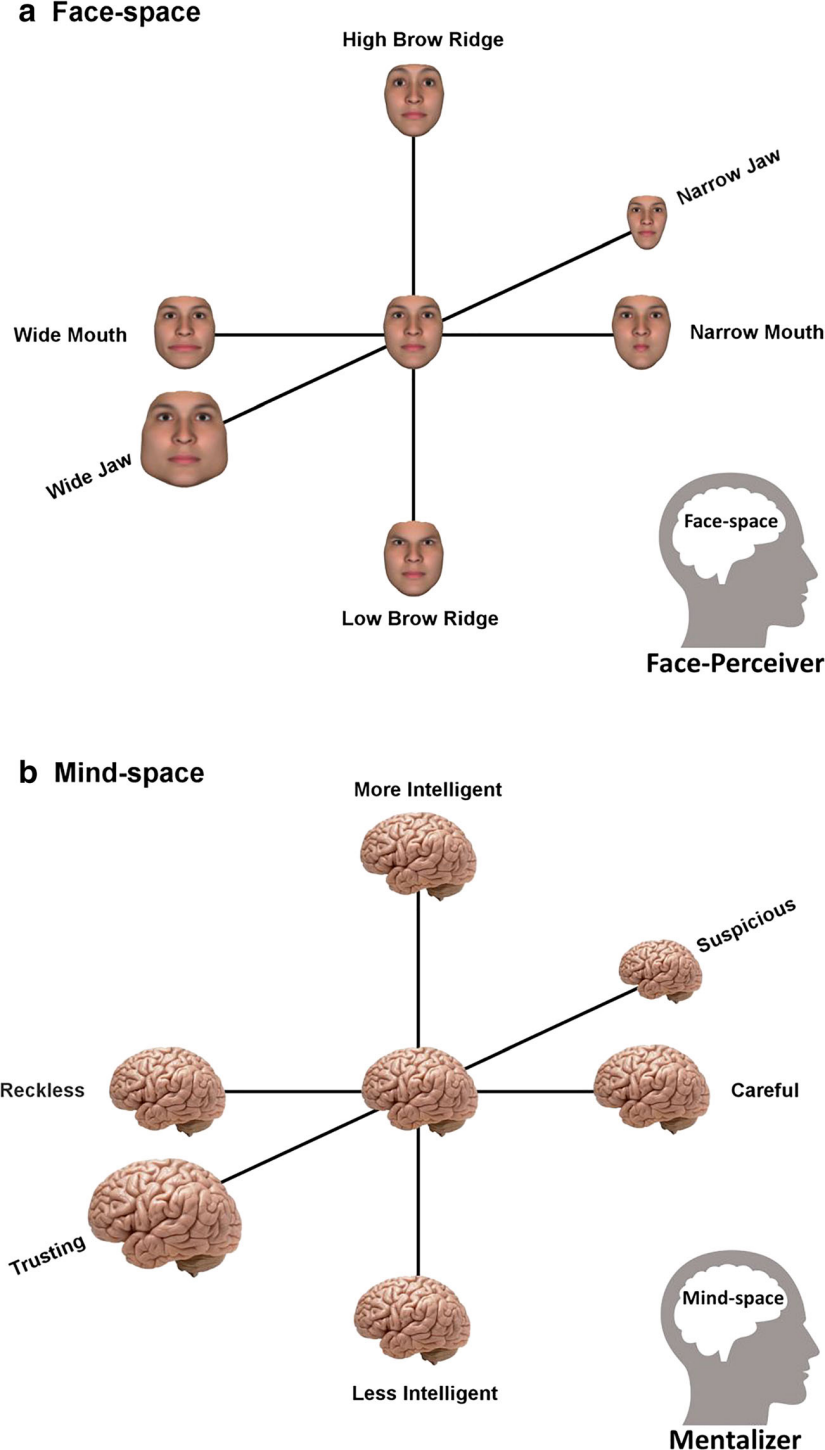
Multidimensional representational spaces: Face-space and Mind-space. In this example of Face-space (**A**), faces are represented on three orthogonal dimensions of brow ridge height, jaw width, and mouth width. In this Mind-space example (**B**), minds are represented on orthogonal dimensions which allow them to be individuated from one another. Dimensions may reflect cognitive abilities (e.g., intelligence), behavioral tendencies (e.g., recklessness), or personality traits (e.g., suspiciousness). (The human brain image is reproduced with permission from Dan Heighton).

We suggest that theoretical and empirical progress on understanding mind representation, and separately the inference of mental states, can be achieved by adopting a Mind-space framework; that minds, like faces, are represented within a multidimensional psychological space (Fig. 1). The Face-space framework was motivated by the lack of a theory that could account for seemingly disparate findings in the face-processing literature, and by the need for a model that would reflect the effect of variance in faces experienced by the individual (Valentine, 1991; Valentine et al., 2016). Face-space is a multidimensional space, the dimensions of which are unspecified but can represent any discriminable aspect of faces, from structural aspects such as nose length to more abstract traits, like attractiveness or trustworthiness (Fig. 1a). In someone’s Face-space, every individual face is represented as a vector along multiple dimensions; the population of experienced faces is normally distributed and the intercept of the axes reflects the dimensional means (Valentine et al., 2016). Although the idea that representations of stimuli are structured along dimensions extends to most percepts, including features of non-social objects such as color, size, or tilt (Thompson & Burr, 2009), Face-space has provided a psychological model to explain a range of empirical findings and acts as a unifying theory of how representations of such complex social stimuli may be structured. Effects explained by the Face-space framework include: why distinctive faces are better recognized than typical faces, even when inverted (Valentine, 1991); why there is an own-ethnicity face recognition bias (Chiroro & Valentine, 1995); perceptual adaptation effects (Jeffery & Rhodes, 2011; Jiang, Blanz, & O’Toole, 2006, 2009; Leopold, O’Toole, Vetter, & Blanz, 2001; Rhodes, Jeffery, Watson, Clifford, & Nakayama, 2003; Webster, Kaping, Mizokami, & Duhamel, 2004); and why children’s face-processing abilities may differ to adults’ (de Heering, Rossion, & Maurer, 2012; Hills, Holland, & Lewis, 2010).

We suggest that a Mind-space framework can overcome current theoretical limitations on mind representation. In common with faces, minds present many dimensions on which they may be similar to, or discriminated from, one another. It is therefore possible to represent individual minds within a multidimensional space, analogous to how faces are represented within Face-space (Fig. 1b). There is no requirement for the axes that represent the space to be orthogonal, meaning that the space can be constructed such that the relationship between axes represents the co-variance between properties of minds encountered in the real world. For example, if a bivariate correlation exists such that one property of minds, suspiciousness, predicts another property, such as aggressiveness, then axes can be constructed such that movement along the suspiciousness dimension causes movement along the aggressiveness dimension. Within such a Mind-space framework, an individual’s representation of another mind can be described as a single vector, or location, in a space determined by multiple axes.

### Representation of the whole cognitive system and variability in mind type

The Mind-space framework allows multiple aspects of mind to be represented within one model; one dimension may represent suspiciousness, another working-memory ability, and another political persuasion. However, this is only necessary if people actually represent those properties of minds that allow them to be differentiated, in addition to the contents of their mental states. Evidence for such representation is provided by examples of ‘recipient design’ – the adaptation of one’s communications to better suit a specific addressee (Blokpoel et al., 2012). For example, several studies using the Tacit Communication Game (Stolk, Noordzij, Verhagen, et al., 2014a; Stolk, Noordzij, Volman, et al., 2014b) demonstrated that communicators modulate their communicative behavior as a function of whether they think they are communicating with someone younger than them (Newman-Norlund et al., 2009; Stolk, Hunnius, Bekkering, & Toni, 2013). The adaptations made by communicators are frequently attributed to the representation of the addressee’s mental states, e.g., beliefs or knowledge (Blokpoel et al., 2012; Newman-Norlund et al., 2009; Stolk, Noordzij, Verhagen, et al., 2014a; Stolk, Noordzij, Volman, et al., 2014b; Stolk et al., 2013). However, modulation of communicative behavior as a function of addressee age suggests that communicators are representing the cognitive processes of the addressee (such as their working memory capacity or inspection time) in addition to their mental states. Similarly, the adoption of ‘elderspeak’ when communicating with older adults, by using slower, shorter sentences (Kemper & Harden, 1999; Williams, Kemper, & Hummert, 1995), likely reflects representations of the memory and processing speed of older adults. Indeed, accurate comprehension of others’ communications can be affected by representations of their linguistic background. The ‘speaker-model’ account of word recognition suggests that listeners disambiguate words with different dominant meanings in British compared to American English by first identifying the speaker’s dialect and then adopting that model for subsequent interpretations (Cai et al., 2017).

Neuroimaging studies have suggested that the medial prefrontal cortex (mPFC), a brain region in the ‘theory of mind network’, may encode information about other people and their personality traits (Hassabis et al., 2014; Heleven & Van Overwalle, 2015, 2018). Suppression effects in the ventral mPFC have been observed with repetition of the same trait (Ma et al., 2014) or person (Heleven & Van Overwalle, 2015). Ma et al. (2014) found suppression effects both for pairs of stimuli that signified the same trait (e.g., honesty + honesty) and for pairs that signified the opposite trait (e.g., dishonesty + honesty). This latter finding holds particular significance for the Mind-space theory, as it implies that traits of others’ minds are represented along dimensions and not categorically (Heleven & Van Overwalle, 2018).

### The relevance of Mind-space to theory of mind

**Fig. 2:**
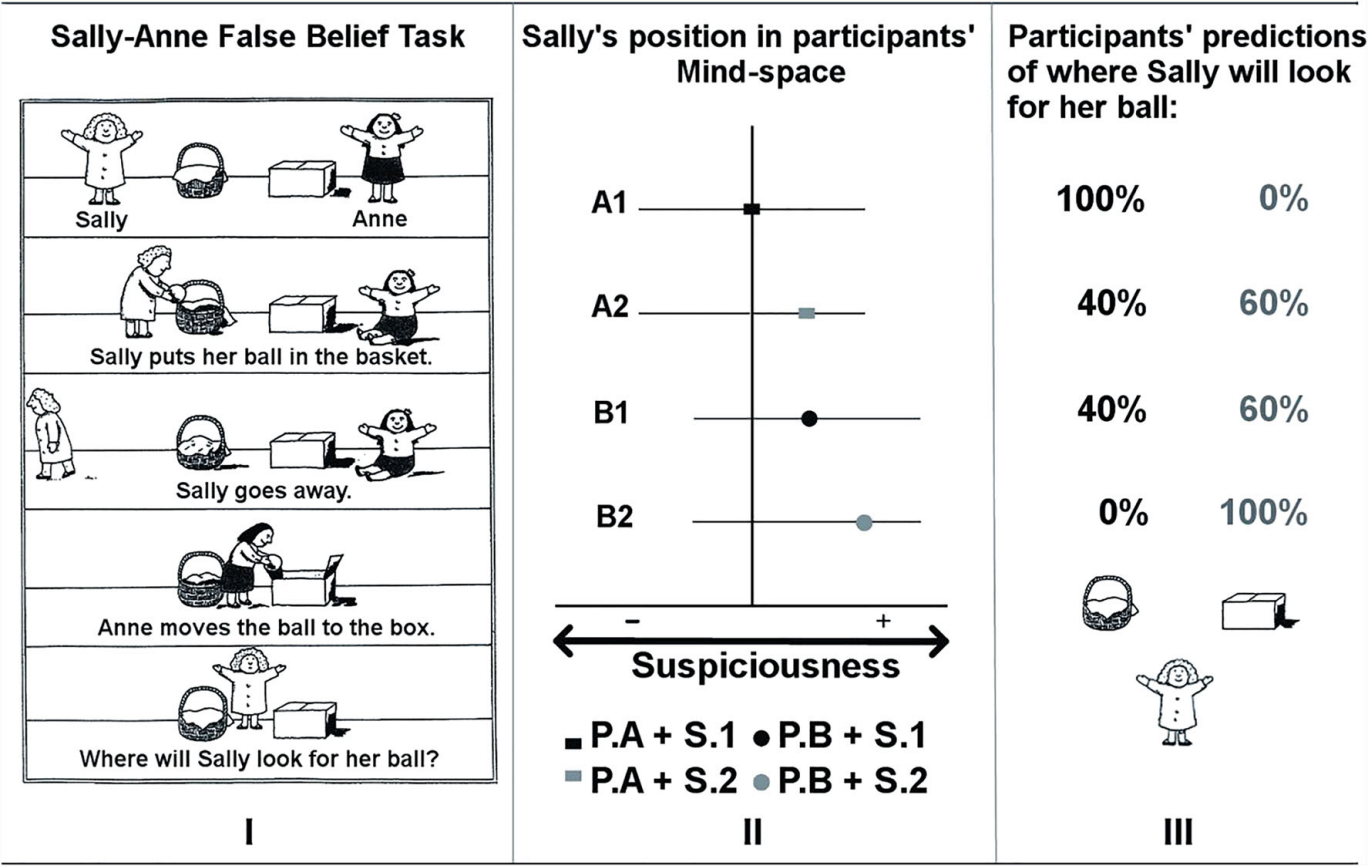
Suspicious minds: How Mind-space explains performance on the Sally-Anne false belief task. In this test of theory of mind (*Panel I*), to respond correctly participants (**P**) must represent Sally’s mental state in the absence of any additional information about her, Anne, or the situation (**S**). In this scenario (Situation 1), an average participant (**P.A; Panel II**) would likely represent Sally at the population mean of suspiciousness in his/her Mind-space, and expect Sally to think that her ball was in the basket where she left it (**Panel III**). The same average participant (**P.A**) in a different situation (**S.2**), having prior knowledge that Sally has high levels of suspiciousness, would represent Sally at a position of high suspiciousness further from the mean. Participant A in Situation 2 might therefore represent Sally as believing that Anne may have moved her ball to the box. Another participant (**P.B**) who has been exposed to an untrustworthy population may, in the absence of any information (**S.1**), have a mean suspiciousness higher than the population average, and, positioning Sally at the mean in his/her Mind-space, similarly represent Sally as believing that Anne may have moved her ball to the box. In Situation 2, having prior knowledge that Sally has high levels of suspiciousness, Participant B would represent Sally further from his/her mean and attribute to Sally the belief that Anne has certainly moved her ball to the box. This example demonstrates how an individual’s representation of Mind-space combines with situational information to influence the inference of another person’s mental state. (Panel 1 reproduced with permission from Frith, 2003).

Mental states are a product of the minds that give rise to them. Accurate and specific inference of the contents of another’s mental states is therefore likely aided by representing multiple features of minds and variability in mind type. For example, theory of mind is commonly tested using a false-belief task such as the Sally-Anne task (Fig. 2, Panel I) (Baron-Cohen et al., 1985). In this task participants are introduced to two characters, Sally and Anne, and are informed that Sally has a ball that she places into her basket before leaving the room. While Sally is away, Anne takes Sally’s ball and places it in her own box. Participants are asked where Sally will look for her ball on her return. This type of paradigm is frequently described as providing the strongest evidence of mental state representation (Baron-Cohen et al., 1985; Dennett, 1978) because successful performance requires the ascription of a false belief: that Sally will act based on a false belief that is inconsistent with where the object actually is and where the participant knows it to be located. Participants are therefore determined to have given a correct answer if they respond that Sally will look in her basket, and an incorrect answer if they respond that Sally will look in Anne’s box. While this task is relatively straightforward, one can imagine that what is deemed a correct answer is likely to change if we know that Sally has high levels of suspiciousness and is likely to suspect Anne has stolen her ball. In this case we may imagine that Sally will first look in Anne’s box to check her assumption that Anne has stolen her ball. In this scenario, a participant who has a dimension of suspiciousness in their Mind-space and who recognizes that Sally is at the extreme end of this dimension is likely to be more accurate when inferring the content of Sally’s mental states than another individual who either does not represent suspiciousness as a property of minds, or who cannot locate Sally accurately along the suspiciousness dimension (Fig. 2).

It can therefore be seen that adopting a multidimensional representational space offers a framework for investigating individual differences in the ability and propensity to represent the properties of other minds, and an explanation of differences in the accuracy and specificity with which the contents of mental states can be inferred. Within the Mind-space framework, the model of a specific other’s mind would serve as a function that takes as its input the context the other is in, and outputs the likelihood of particular mental states. In statistical terms, one can represent this as the probability of a particular mental state given a particular context and the position of the target mind within an individual’s Mind-space. Individual differences in the representation of other minds, and in the accuracy of mental state inference, would therefore be due to one or more of the following factors:

1. Fundamental features of the architecture of an individual’s representation of Mind-space such as the complexity of the representational space in terms of the number of dimensions and representation of the co-variance between dimensions, or the ‘granularity’ or level of detail represented in each dimension.
2. The accuracy with which one can locate a target mind within one’s Mind-space on the basis of a sample of behavior.
3. The propensity of an individual to represent minds within Mind-space, and the degree of effort expended in locating a target mind within Mind-space with a high degree of precision.
4. The accuracy of the mapping between position in Mind-space and specific mental states (e.g., the mapping from Panel 2 to Panel 3 in Fig. 2), and the propensity to use position in Mind-space when making a mental state inference.

### The self, metacognition, and Mind-space

The question of whose mind is modelled as the default – i.e., the mind that is used to ascertain the probability of particular mental states given situational information only – has long been a topic of debate within the theory of mind literature. One prominent account, the Simulation Theory, posits that one uses one’s own mind as this default, to run a simulation that outputs the probability of specific mental states, the most likely of which is then ascribed to the target (Carruthers & Smith, 1996). In this account, egocentric effects are likely to be observed; one attributes the mental state one’s own mind would generate if in the same situation as the target. Under the Mind-space framework, however, if one has the propensity to use position in Mind-space when inferring mental states, one does not use one’s own mind as a model of others. Rather, one represents a target mind’s position in Mind-space, or in the absence of any individuating information (i.e., for an anonymous protagonist), likely assumes the mind to be in the center of Mind-space (representing the population average on each dimension of Mind-space).

The distance between the center of an individual’s Mind-space and where they believe their own mind to be located within Mind-space is likely to vary across individuals. Some individuals would judge themselves to be average on some or all dimensions, while others would judge themselves to be more extreme. We use the term ‘metacognitive accuracy’ to refer to the degree to which an individual can accurately locate themselves in Mind-space; those with high metacognitive accuracy would, for example, be able to judge their IQ relative to the rest of the population, whereas someone with low metacognitive accuracy would either over- or under-estimate their IQ relative to the rest of the population.

The distance between the center of an individual’s Mind-space and where they judge their own mind to be in Mind-space is likely to have important implications for how accurately they can infer the mental states of an anonymous target; furthermore, the effect of this distance on the accuracy of mental state inferences will be moderated by the individual’s metacognitive accuracy. The privileged access to one’s own mental states is likely to result in extensive and enduring mappings between the location one believes oneself to occupy in Mind-space and the mental states experienced in particular situations, due to the fact that one receives more data about one’s own mental states than others’ mental states, and mappings are likely to be less variable than those provided by experience of a variety of other individuals. Thus, an individual who locates their own mind in the center of their Mind-space can use their own mind as a model for an anonymous target mind (which is most likely to be also in the center of their Mind-space), or for minds they judge to be similar to their own (i.e., estimated also to be in the center of their Mind-space). Accuracy when inferring the mental states of such target minds will therefore depend on two factors: (1) The individual having good metacognitive accuracy and therefore truly being in the center of their Mind-space; and (2) the individual accurately locating targets within Mind-space (and therefore the targets are truly in the center of their Mind-space). Providing these two conditions are satisfied, good accuracy is afforded by the increased accuracy of the mappings between location in Mind-space and the probability of particular mental states resulting from the privileged access the individual has to their own mental states. If an individual has good metacognition but does not locate their own mind at the center of their Mind-space, then their own mind is not a good model for an anonymous target mind (who would be located at the center); however, if they can accurately locate targets within their Mind-space then their own mind will act as a good model for targets similar to the self.

In contrast, if the individual has poor metacognitive accuracy but can accurately locate others in Mind-space, then they are likely to make inaccurate inferences concerning the mental states of targets whom they either believe to have a mind like their own, or targets who actually do have a mind similar to their own. Furthermore, when poor metacognitive accuracy but an intact ability to locate others within Mind-space is combined with accurate mappings between locations in Mind-space and mental states, then the individual would exhibit a decreased ability to predict the likelihood of their own mental states – a situation likely to result in disorders characterised by an atypical sense of self, self-delusions, or a reduced sense of agency.

Non-metacognitive aspects of the self may also impact upon one’s Mind-space. For example, an individual very high on trait agreeableness may be less likely to attribute negative attributes to others, or attribute less extreme negative attributes. This would result in a Mind-space where negative attributes are skewed towards low scores, have low mean values or granularity, or co-variances are inaccurately represented. Similarly, individuals who tend to attribute behavior to aspects of the situation rather than the characteristics of the target’s mind may be slower to: (1) construct a Mind-space; (2) learn to locate targets within Mind-space in general; or (3) learn to locate a specific target within their Mind-space.

### Relationship to existing theories

When considering the relationship between the current proposal and existing theories it is first worth acknowledging what is not novel about the proposal. Most obviously, it is clear that trait models have previously been used in psychology, notably within the field of personality where dominant models suggest that variance in personality can be explained using a model with five or six trait dimensions (Ashton & Lee, 2007; Goldberg, 1990; McCrae, 1989). Of more relevance to Mind-space are existing dimensional models of how we represent individuals, groups, or other agents. For example, Gray, Gray, and Wegner (2007) suggested that judgments regarding other agents’ (e.g., children, robots, supernatural beings) ability to feel pain, emotions, have personalities, etc. can be accounted for by a two-dimensional model of whether they are capable of having experiences, and whether they have agency. Perhaps closer to the concept of Mind-space is the work of Fiske and colleagues (Fiske, Cuddy, & Glick, 2007), who have convincingly demonstrated that the dimensions of warmth and competence explain a large degree of the variance in how individuals and groups are perceived. It is therefore clear that the idea that humans can represent other humans (and non-human agents) on trait dimensions which can be described by a reduced set of dimensions or factors is not novel.

The novel feature of the Mind-space proposal is that it explains how variance in *representing minds*; specifically, variance in the structural properties of the multidimensional space within which minds are represented, can explain individual differences in *the ability to make mental state inferences*. In this context, it is important to consider how it relates to the work of Tamir and Thornton (Tamir & Thornton, 2018; Tamir, Thornton, Contreras, & Mitchell, 2016), who have developed an independent proposal relating trait representation to mental states and actions.

Tamir and Thornton’s primary aim is not to explain individual differences in the ability to make mental state inferences, but rather to identify the information used to make social predictions and how it is represented. Accordingly, they posit the existence of a multilayered dimensional framework where the layers correspond to others’ actions, mental states, and traits, and each of these layers can be characterized on the basis of three dimensions. They put forward an interesting account of how transitions between these layers may allow the prediction of social behavior, an account that is compatible with several existing dimensional theories of person and agent perception (e.g., Fiske et al., 2007; Gray et al., 2007).

As mentioned above, this account does not address individual differences (in the dimensional structure of the multi-layered framework, the ability to locate a target mind accurately within it, or the propensity to do so). Furthermore, the nature of the mental state representations is very different in the Tamir and Thornton and Mind-space frameworks. To illustrate, the dimensions used to represent mental states in the Tamir and Thornton framework are rationality, social impact, and valence; and these can be used to encode concepts such as emotions (disgust) and states of mind (intoxicated, weary, fatigued), or to distinguish between mental state types (opinion, belief, thought). Under the Mind-space framework, however, it is minds, not mental states, that are represented dimensionally. Mental *states* are *not* represented dimensionally because the Mind-space framework attempts to explain variance in the ability to infer the content of specific mental states, and in many cases this content is unlikely to be represented in a dimensional structure. For instance, in the case of the Sally-Anne example (Fig. 2), propositional attitudes such as ‘John believes that Sally will look for her ball in her basket’ and ‘John believes that Sally will look for her ball in Anne’s box’ are very different, yet presumably would be located in exactly the same location in the Tamir and Thornton framework, as that framework distinguishes between mental state types (e.g., ‘belief’ vs. ‘desire’), but does not encode mental state content.

Correct inference of specific mental state representations relies on consideration of situational factors, which are currently outside the Tamir and Thornton framework. However, recognition of the importance of situational factors prompts consideration of how the hypothesized role for Mind-space in the inference of mental states can be reconciled with recently developed computational models of mental state inference that describe how mental states might be predicted on the basis of the situation. We suggest that the addition of Mind-space terms to these computational models of mental state inference may significantly improve their predictive validity, and allow them to be tailored to specific individuals or groups.

An example of such a model is the Bayesian Theory of Mind (BToM) model of Baker et al. (2017), which models the computational basis of ‘core mentalizing’: meta-representation of the percepts, desires, and beliefs of a rational agent inferred from their actions in a given physical spatial environment. In the BToM framework, it is assumed that the agent updates its beliefs based on percepts and prior knowledge, and acts rationally to achieve its desires with maximum efficiency and minimum cost. Inference of the agent’s beliefs and desires is achieved through inversion of a generative model which describes how mental states cause actions. The generative model is conditioned on observed actions, and representation of unobserved mental states (percepts, beliefs, desires) is thought to be a result of Bayesian inference. The BToM model has been shown to be a successful model of human mental state inference (at least in constrained environments with a limited set of possible desires and beliefs, Baker et al., 2017). However, although BToM is a successful model of how such inference may work in general, by incorporating the position of a specific agent in a particular individual’s Mind-space one can further constrain the set of inferences likely to be made about the agent’s mental states by that individual (Jacob & Jeannerod, 2005). Furthermore, one can explain why that individual’s inference differs from that of another individual, and therefore why one individual is more or less accurate than another. Inclusion of the Mind-space framework within Bayesian generative models of mental state inference may therefore increase their specificity with respect to particular individuals. In addition to increased specificity, modelling of an agent’s position within an individual’s Mind-space, particularly on dimensions such as intelligence, attention to detail, and perseveration, is likely to explain the degree to which the individual expects the agent to update the content of its mental states as a function of experience within a dynamic system.

For example, the probability of a particular mentalizer inferring that an individual target mind holds a certain mental state is a function of the prior probability of:

- that mental state in general;
- the probability of the mental state conditional upon the situation the target is in;
- and the position of the target in the mentalizer’s Mind-space.

**Fig. 3:**
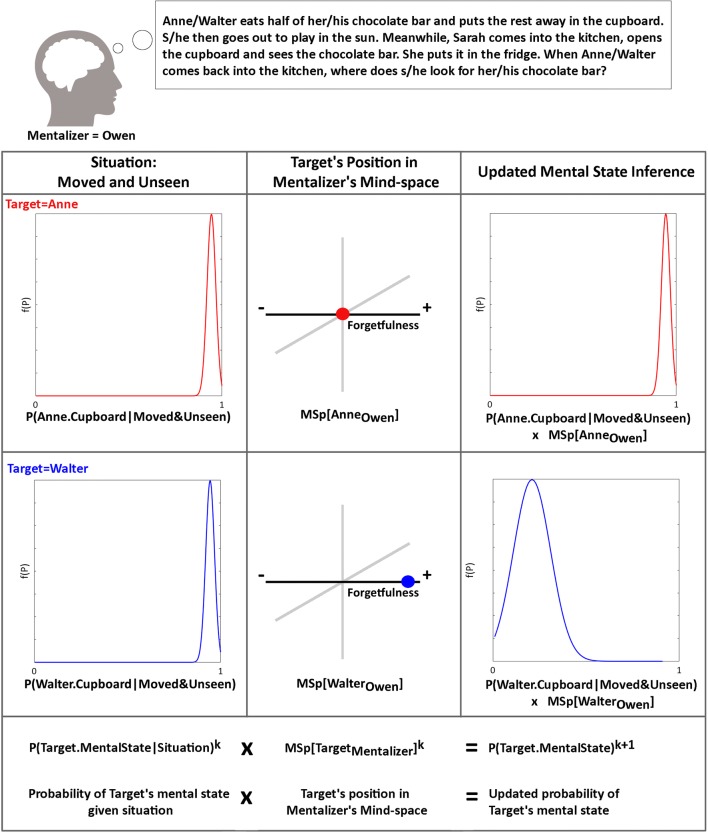
The relationship between situation, Mind-space, and mental state inference. An example of how the situational factors and location of a target in a mentalizer's Mind-space predict the probability of the mental state content inferred (k = sampling time). Based only on the situational factors, Owen (the mentalizer) predicts that both Anne and Walter are likely to look for their chocolate in the cupboard. Considering their respective positions in Owen's Mind-space on the forgetfulness dimension, Owen revises his prediction for Walter, who is very forgetful and therefore less likely to remember he left the chocolate in the cupboard.

The relative influence of situational factors and the target’s position in the mentalizer’s Mind-space on the posterior estimate of the probability of the target’s mental state will be determined by the precision of the prediction each affords. For example, if the target is being chased by a bear then one may make a very precise prediction as to their mental state on the basis of the situation they are in, whereas the prediction based on their position in the mentalizer’s Mind-space is likely to be less precise. In this situation, the posterior prediction of the target’s mental state will be governed more by the context than by their position in the mentalizer’s Mind-space. There may be other contexts where the situation allows a less precise prediction of the target’s mental state, and position in Mind-space a more precise prediction. In this case, the mentalizer’s posterior prediction would be based more on the target’s position in the mentalizer’s Mind-space than the situation the target is in. Note however, that even if it is the case that position in Mind-space affords a precise prediction of the relevant mental state in principle, it may still be the case that the mentalizer has an imprecise representation of the location of the target in their own Mind-space. As a consequence, the prediction of the probability of a certain mental state given a target’s position in Mind-space will also be imprecise (see Fig. 3).

## Predictions and implications of the Mind-space framework

The development of Face-space is thought to be experience-dependent. The space is optimized for the population of faces to which one has been exposed so that the population of faces one encounters most often can be efficiently individuated (Balas, 2012; Valentine, 1991; Valentine et al., 2016). We suggest that Mind-space is similarly experience-dependent, such that the structure of Mind-space reflects the population of minds to which an individual has been exposed. One’s developmental experience of different minds would therefore determine the number and type of possible dimensions, and the co-variance between dimensions in Mind-space, in order to enable efficient representation and individuation of the type of minds frequently encountered (Astuti, 2015). Once an individual has constructed their Mind-space then they must learn the mean and variance of each mind they encounter on each of the multiple dimensions and revise the structure of their Mind-space where necessary.

Such an optimization process within Face-space is thought to be responsible for the own-ethnicity advantage to face recognition (Chiroro & Valentine, 1995; Valentine & Endo, 1992), whereby one is better able to individuate faces from one’s own ethnic background than those from another ethnic background. It is argued that the number, type, co-variance, and scaling of dimensions are optimized according to the population of faces most commonly experienced (typically from one’s own ethnicity), and therefore this space is not optimized to individuate faces drawn from another population (i.e., from a set of other-ethnicity faces), which require a different Face-space structure for optimal individuation. Although experience requiring the individuation of other-ethnicity faces improves this ability, it is interesting to note that this type of experience results in a small decrement in the ability to recognize own-ethnicity faces (Chiroro & Valentine, 1995), presumably as Face-space is no longer perfectly optimized for either population but instead optimized for best performance across the two populations of faces (Valentine et al., 2016).

An analogous process within Mind-space would result in poor models of minds that deviate from the population of minds that one normally encounters. Indeed, Happé and Frith (1996) suggested that children who grow up in abusive or neglectful homes and who are later diagnosed with Conduct Disorder may have developed a model of “nasty” minds, where they overestimate the tendency of others to have minds characterized by aggression, deceitfulness, and a lack of empathy. This model of nasty minds may cause them to be more likely to react with aggression and suspicion when dealing with others, even in the absence of aggression directed towards them. In a similar vein, Frankenhuis and colleagues discuss why those who experienced early life stress such as violence in the home can be faster to identify threat and anger, and better at inferring social dominance and group hierarchy, than those without such developmental experience (Frankenhuis & de Weerth, 2013; Frankenhuis & Del Giudice, 2012; Frankenhuis, Panchanathan, & Nettle, 2016). Less pathologically, optimization of Mind-space for one’s own social group may lead to poor appreciation and understanding of the points of view of those who differ in age, political outlook, culture, or level of education from one’s own group, and/or a failure of negotiation when dealing with unfamiliar others.

Inter-group contact has been repeatedly demonstrated to improve the ability of different groups to understand each other’s views, reduce stereotyping and increase individuation (Brambilla, Ravenna, & Hewstone, 2012; Bruneau & Saxe, 2012; Harwood, Hewstone, Paolini, & Voci, 2005; Schmid, Ramiah, & Hewstone, 2014), and this may be because such experience allows the modification of Mind-space for efficient representation and individuation of minds dissimilar to those experienced throughout one’s developmental history. Indeed, the development and use of stereotypes may reflect poor calibration of Mind-space and a resultant lack of individuation for members of groups other than one’s own. If Mind-space works in the same way as Face-space, then the prediction would be that recalibration of Mind-space in response to a distinct population of minds would also result in a small reduction in ability to model the original population of minds, if optimization of Mind-space for both populations of minds results in a sub-optimal space for each independent population (Chiroro & Valentine, 1995; Valentine et al., 2016). A restructuring of Mind-space may serve as a psychological or neurological marker of the reduction in inter-group conflict following inter-group contact.

The experience-dependent nature of Mind-space, and the fact that the accuracy of any particular mental state inference will depend on the quality of the model of a particular mind, means that it becomes less meaningful to talk of an individual or group’s ‘theory of mind ability’ in general terms. A specific individual may be able to infer the contents of a particular target’s mental states very well, yet be poor at inferring those of a different target. This can be demonstrated empirically; although typical individuals may exhibit a high degree of accuracy when inferring the mental states of other typical individuals, they are less good at recognizing the emotions (Brewer et al., 2016; Macdonald et al., 1989; Volker, Lopata, Smith, & Thomeer, 2009) and mental states (Edey et al., 2016) of individuals with Autism Spectrum Disorder. To some extent however, a degree of general ‘theory of mind ability’ (whether good or poor) might be expected due to individual differences in the propensity to model other minds before inferring their mental states, or individual differences in social attention (Chevallier, Molesworth, & Happé, 2012) or social learning (Cook, den Ouden, Heyes, & Cools, 2014), which may impact the speed and quality of learning required to develop Mind-space itself and/or accurately locate an individual target mind within Mind-space. Thus, although the ability to represent minds and the propensity to do so are logically distinct, a greater propensity to represent minds may provide more opportunity for experience-dependent tuning of one’s Mind-space, which, given an appropriate learning environment, would increase the accuracy of mind representation and mental state inference.

Some of the strongest evidence for the experience-dependent and dimensional aspects of Face-space comes from adaptation effects. Face adaptation occurs when exposure to faces at extreme ends of a dimension, such as attractiveness (Rhodes et al., 2003), gender (Webster et al., 2004), or contractedness (Jeffery & Rhodes, 2011), shifts the mean of that dimension such that stimuli originally perceived as neutral subsequently appear further from the adapting face. For example, prolonged exposure to a very wide face will mean that other faces are perceived as narrower than before the exposure to the wide face. There is some indirect evidence that adaptation may also occur in Mind-space; Xiang and colleagues (2013) demonstrated that exposure to generous or unfair offers in an Ultimatum Game affected subsequent rejection rates and mood ratings for fair, neutral and unfair offers. Directly testing for adaptation effects in Mind-space would provide a strong test of whether minds are represented along dimensions (Heleven & Van Overwalle, 2018; Ma et al., 2014), rather than categories, and whether experience affects the structure of Mind-space.

### Typical and atypical development of Mind-space

We have suggested that the development of Mind-space is experience-dependent. Typical developmental effects in the ability to represent minds and accurately infer the content of mental states may reflect the formation of a higher-dimensional Mind-space, more appropriate weighting of dimensions, and/or an increasing ability or propensity to locate individuals within Mind-space. Indeed, considering atypical development of Mind-space provides for the establishment of further sources of individual differences in mental state inference. Over development, one must learn the relative importance of different dimensions of Mind-space in determining mental states in particular contexts, and how variance in these dimensions predicts variance in mental states. Atypical experience may lead to atypical mental state inferences even when the target is located correctly in a typical Mind-space. For example, if a child grew up in a family with a depressed parent who exhibited atypical depression-related mental states (i.e., atypical within the population of depressed individuals), then they may learn an atypical model of how position on the depression dimension of Mind-space predicts the likelihood of specific types of mental state. If they subsequently encounter a second depressed individual, who they correctly locate on the depression dimension in their Mind-space, then if they apply this atypical ‘Mind-space to mental state’ model to the second depressed individual they will make an incorrect inference regarding their mental state. Additionally, if mind representation is culturally acquired, then the Mind-space framework is sufficiently flexible to account for cultural differences in how minds are represented. Theories of minds change across cultures (Lillard, 1998; Perez-Zapata, Slaughter, & Henry, 2016; Shahaeian et al., 2011), and perhaps across historical time, and therefore any psychological model of how minds are represented needs to account for varying concepts of mind.

Mind-space provides a framework for investigating the development of advanced social skills; for example, the ability to quickly extract diagnostic information to locate someone within Mind-space. Conversely, the Mind-space framework may shed light on the social impairments which are a transdiagnostic trait of many psychiatric and neurodevelopmental disorders, including autism, depression, eating disorders, and personality disorders (Happé, 1994; Preißler, Dziobek, Ritter, Heekeren, & Roepke, 2010; Russell, Schmidt, Doherty, Young, & Tchanturia, 2009; Wang, Wang, Chen, Zhu, & Wang, 2008). Under this framework social impairments may reflect: (1) an atypical representation of Mind-space (for example, the paranoia observed in schizophrenia (Drake et al., 2004) could reflect a misaligned, over-weighted, or otherwise atypical dimension representing others’ hostility); (2) a decreased propensity to model other minds; or (3) a fundamentally altered learning system that results in decreased generalization of learning (e.g., Plaisted, 2001), or a reduced influence of priors (Pellicano & Burr, 2012), which impacts the updating of Mind-space from experience. For example, it has been claimed that individuals with autism show insufficient generalization of their learning (Plaisted, 2001). As a consequence, autistic individuals may be too specific in their mental models, failing to generalize across instances to develop population-based representations of Mind-space. Conversely, some of the social difficulties encountered by individuals diagnosed with psychiatric conditions may be caused by a failure of typical individuals to be able to develop an accurate model of atypical minds (Brewer et al., 2016; Edey et al., 2016; Sasson et al., 2017).

## Concluding remarks

In this article we sought to address an impasse in the theory of mind literature, specifically the inability of current frameworks to characterize individual differences in theory of mind ability, and to introduce a framework within which all aspects of minds can be represented. We have suggested that the adoption of a Mind-space framework where minds are represented within a multidimensional space – similar to that which has been so successful in providing a unifying theoretical framework for the study of faces – would achieve both aims. Mind-space represents a psychological model of a representational structure involved in the representation of minds, which may also explain variance in the accuracy of mental state inference. It considers how individuals build models of other minds, and suggests that there may be substantial variance in the accuracy of mental state inference within an individual based on the quality of their representation of the target mind. Future work can determine whether analogous effects to those in the face processing literature explained by Face-space can be observed for mind representation by adopting the Mind-space framework. Findings equivalent to the own-ethnicity bias and perceptual adaptation seen in faces would explain much about how inter-group conflict may be generated, maintained, and reduced. We hope that this introductory sketch of Mind-space is a first step towards an understanding of individual differences in the representation of whole cognitive systems, where minds are recognized as complex multidimensional stimuli. It should be noted, however, that even if minds are not represented in a multidimensional space, the ability and propensity to represent another’s mind is still likely to be an important source of individual differences in the accuracy of mental state inference.
